# Access to general practice for people with intellectual disability in Australia: a systematic scoping review

**DOI:** 10.1186/s12875-022-01917-2

**Published:** 2022-11-29

**Authors:** Bradley Shea, Jodie Bailie, Sally Hall Dykgraaf, Nicola Fortune, Nicholas Lennox, Ross Bailie

**Affiliations:** 1 Sydney Medical School, The University of Sydney, Sydney, NSW Australia; 2 University Centre for Rural Health, The University of Sydney, Lismore, NSW Australia; 3 Centre for Disability Research and Policy, The University of Sydney, Sydney, NSW Australia; 4 Rural Clinical School, Australian National University, Canberra, ACT Australia; 5 Centre of Research Excellence in Disability and Health, University of Melbourne, Carlton, VIC Australia; 6 Queensland Centre for Intellectual and Developmental Disability, University of Queensland, Brisbane, QLD Australia

**Keywords:** Primary care, Intellectual disability, Family medicine, Learning disability, Access

## Abstract

**Background:**

People with intellectual disability experience inadequate access to general practice and poorer health outcomes than the general population. While some access barriers have been identified for this population, these studies have often used narrow definitions of access, which may not encompass the multiple dimensions that influence access to general practice. To address this gap, we conducted a scoping review to identify factors impacting access to general practice for people with intellectual disability in Australia, using a holistic framework of access conceptualised by Levesque and colleagues.

**Methods:**

This scoping review followed Joanna Briggs Institute methodology and was guided by the Preferred Reporting Items for Systematic Reviews and Meta-Analyses Extension for Scoping Reviews. Medline (Ovid), Scopus, CINAHL, Informit and PsycINFO databases were searched. Screening, full-text review and data extraction were completed by two independent reviewers, with consensus reached at each stage of the study. Data were extracted, coded and synthesised through deductive qualitative analysis – using the five corresponding conceptual dimensions within Levesque and colleagues’ theoretical framework of access, which incorporate both supply-side features of health systems and services, and demand-side characteristics of consumers and populations.

**Results:**

The search identified 1364 publications. After duplicate removal, title and abstract screening and full-text review, 44 publications were included. Supply-side factors were more commonly reported than demand-side factors, with the following issues frequently identified as impacting access to general practice: limited general practitioner education about, and/or experience of, people with intellectual disability; communication difficulties; and inadequate continuity of care. Less frequently included were factors such as the health literacy levels, promotion of general practice services and availability of complete medical records.

**Conclusions:**

This is the first scoping review to assess access barriers for people with intellectual disability using a comprehensive conceptualisation of access. The findings highlight the need for increased efforts to address demand-side dimensions of access to general practice and offer a basis for a balanced portfolio of strategies that can support recent policy initiatives to enhance access to care for people with intellectual disability.

**Supplementary Information:**

The online version contains supplementary material available at 10.1186/s12875-022-01917-2.

## Introduction

Access to primary care is vital for meeting health needs and improving health outcomes [1, 2]. From a human rights perspective, high-quality primary care should be accessible to all people, regardless of background, socioeconomic situation or personal circumstance [3]. However, primary care access is not always equitable and, as described by the Inverse Care Law [4], those with the greatest need, paradoxically, often receive the poorest health care.

This is frequently the case for people with intellectual disability, who are known to experience greater rates of multi-morbidity [5]; premature and potentially avoidable death [6]; and potentially preventable hospitalisation [7] compared with the general population. Furthermore, many have high levels of undetected and unmanaged health needs [8, 9]. Inadequate primary care access is thought to contribute to these inequitable health outcomes [7, 10].

Strategies to improve access to primary care for people with intellectual disability are likely to be most effective if factors that affect access and create barriers can be identified [11]. Internationally, known barriers to primary care access for people with intellectual disability include communication difficulties [12–22], inexperienced or inappropriately trained staff [12–19, 21–25], inadequate health service integration and continuity of care [12–17, 22, 26–28], the associated costs of health care [15–17, 22, 29], and a perceived lack of time during appointments [13, 14, 17, 21, 24–27, 30]. However, these studies have often used narrow definitions of access, which may not encompass the multiple dimensions that influence access to primary care.

In Australia, general practitioners (GPs) provide the majority of primary care. People can choose their preferred general practice, and GP services are subsidised under Australia’s government health insurance scheme, Medicare [31]. Despite this, Weise and colleagues have shown that universal access to general practice for people with intellectual disability in Australia is not a reality, and more work is required to identify access barriers [32].

**Fig. 1:**
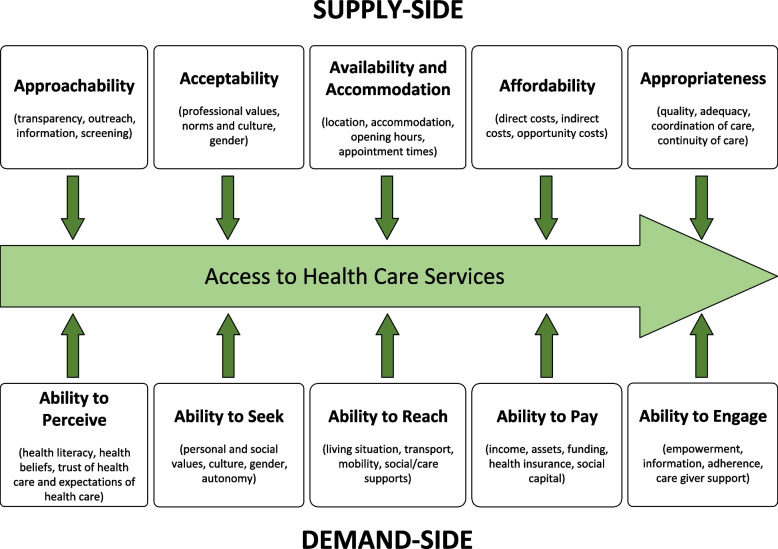
Conceptual framework of access to health care, adapted from Levesque et al. [33]

The Conceptual Framework of Access to Healthcare published in 2013 by Levesque and colleagues [33]*,* take a multidimensional approach, recognising that access occurs through interactions between ‘supply-side’ features of health systems and services, and corresponding ‘demand-side’ characteristics of consumers and populations (Fig. 1). In this conceptualisation, access is defined primarily as an ‘opportunity’ to reach and obtain appropriate health care services, relative to perceived need. This results from the interplay between individual, collective, social and environmental attributes of health care users and corresponding characteristics of health care providers, organisations and systems, across five dimensions. Levesque’s framework has been widely used to examine access to health care for marginalised groups in Australia [34, 35] and internationally [36, 37].

To date, such a holistic conceptualisation of access has not been used with regard to people with intellectual disability accessing general practice in Australia. This review addresses this research gap by assessing the published literature against the Levesque et al. framework [33] to identify supply- and demand-side factors impacting general practice access for Australians with intellectual disability.

## Methods

Due to the broad nature of the research question, the authors employed a scoping review methodology following the Joanna Briggs Institute (JBI) approach [38]. Reporting was guided by the Preferred Reporting Items for Systematic Reviews and Meta-Analyses Extension for Scoping Reviews (PRISMA-ScR) checklist [39]. Neither critical appraisal nor risk of bias assessment of identified publications were conducted, consistent with JBI methodology. All methods were carried out in accordance with relevant guidelines.

### Stage 1: research question

The research question was: *“What factors impact access to general practice for people with intellectual disability in Australia?”*

### Stage 2: relevant literature identification

An initial search of MEDLINE and Google Scholar was conducted by BS and JB to identify studies on the topic and to create a list of relevant search terms. A full search strategy for MEDLINE was developed in consultation with an academic librarian, and with clinical and research experts in the fields of intellectual disability and health services research; the final MEDLINE search strategy can be found in additional file 1. Database searches were conducted in MEDLINE, CINAHL, Scopus, PsycINFO and Informit on 2 February 2022.

### Stage 3: study selection

Identified publications were uploaded into Covidence™ [40], a web-based review platform, and duplicates removed. Two independent reviewers (BS and JB) conducted title and abstract screening using pre-defined inclusion and exclusion criteria (Table 1), following a pilot test. The same two reviewers independently completed full text screening. Percent agreement for study inclusion was 84% for abstract review and 85% for full text review. Discrepancies in both abstract and full text review were resolved through discussion and consensus.

**Table 1:** Inclusion and exclusion criteria

Inclusion Criteria	Exclusion Criteria
1. Population: People with intellectual disability, defined as permanent decreased intellectual function, present during developmental periods, before age 18. People with cerebral palsy, autism or other neurodevelopmental disorders are only included if they have a co-existing intellectual disability. 2. Concept: Determinants of access to primary care, which encompasses features of services and characteristics of users that may act as barriers or enablers to primary care access. 3. Context: Fee-for-service general practice, Government-managed general practice and Aboriginal Community Controlled Health Services. 4. Types of evidence sources: Published literature, including quantitative, qualitative, mixed methods study designs and commentaries.	1. Was published before September 1978, when primary health care was outlined by The Declaration of Alma-Ata. 2. Full text is not published in English. 3. Publication is a report of a research protocol, conference abstract or book review.

### Stage 4: data extraction (data charting)

Data were extracted independently by two reviewers (BS and JB) using a data extraction tool created in Covidence™. This included the methodological and design characteristics of each data source, as well as factors impacting general practice access (Additional file 2). The two reviewers (BS and JB) independently performed pilot data extraction on a random sample of five publications and subsequently refined the data extraction tool to include ‘not specified’ options for setting, jurisdiction, rurality and intersectionality. No other changes were made to the data extraction tool after piloting. Where extracted data differed between reviewers, consensus was reached through discussion.

### Stage 5: data analysis and synthesis

Using extracted data, deductive qualitative analysis [41] was conducted to identify and categorise factors impacting general practice access. These factors were mapped to the Levesque et al. [33] dimensions of access. Both reviewers discussed and cross-checked this mapping, and reflected on and discussed emerging factors of the analysis to ensure consistency and conceptual clarity.

## Results

### Search results and study selection

**Fig. 2:**
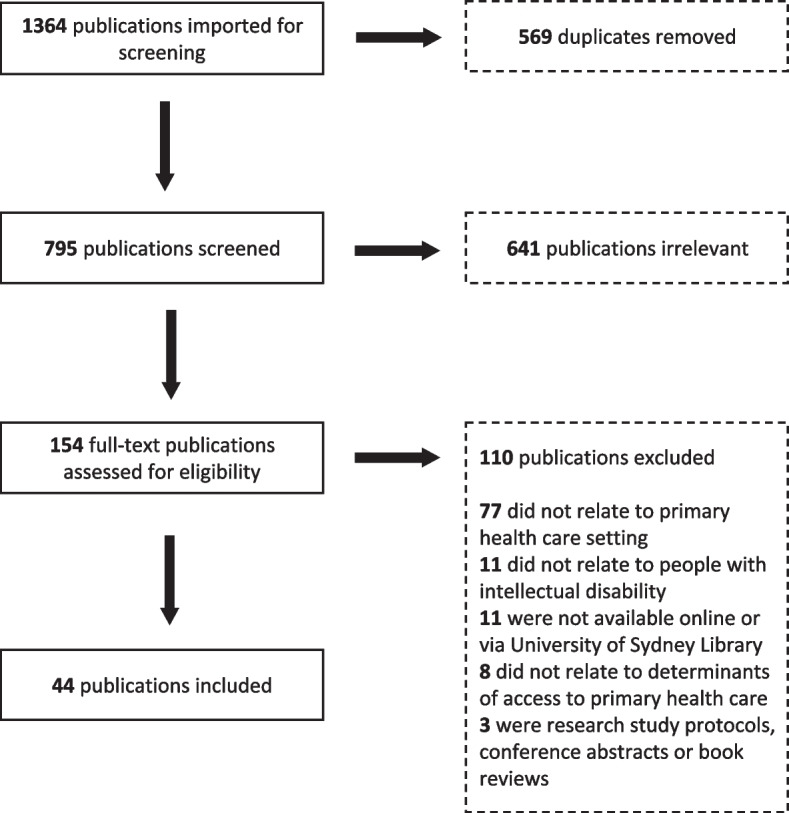
Preferred Reporting of Items for Systematic Reviews and Meta-Analysis Extension for Scoping Reviews (PRISMA-ScR) selection of sources of evidence flow diagram

The search identified 1364 publications. After duplicate removal, title and abstract screening and full-text review, 44 publications were included (Fig. 2).

### Characteristics of included studies

Characteristics of the 44 included publications are displayed in Additional files 3 and 4. Most were empirical research (*n* = 32) with qualitative study designs (*n* = 21). None of them indicated the type of general practice setting: for example, either fee-for-service, government-managed general practice, or Aboriginal Community Controlled Health Services. Most did not specify the rurality of the study setting (*n* = 32), or the study jurisdiction (*n* = 16). Of the publications that specified location, most focused on general practice in Queensland (*n* = 11). However, this is most likely because more than half of the publications (52%, *n* = 23) were authored by a clinical research expert (NL) based in Queensland.

### Factors identified and corresponding dimensions

Results are synthesised below and summarised in Table 2. Identified factors have been categorised according to which Levesque et al. [33] dimensions they align with. Overall, there were 94 instances of supply-side factors being identified across 39 publications, and 66 instances of demand-side factors were identified across 35 publications.

**Table 2:** Factors identified in our study according to the Levesque et al. [33] supply- and demand-side determinants of access

Supply-side determinants	Demand-side determinants
**Approachability** (*4 publications)* - The promotion of general practice services, including targeted preventive health assessments. - GP awareness of targeted preventative health assessments. - The need for clinical information systems that can identify people with intellectual disability, so it is easy to invite, recall and remind people to participate in general practice.	**Ability to perceive** ***(****11 publications)* - Generally low health-literacy levels of people with intellectual disability, their family and support workers. - Generally low educational levels, which reduces the ability to access health information for some people with intellectual disability. - Need for targeted health promotion for people with intellectual disability.
**Acceptability** *(28 publications)* - GP education and experience of treating people with intellectual disability. - Perceived discrimination, insensitivity or negative attitudes from GPs in the health care of people with intellectual disability. - Provision of reasonable adjustments to facilitate access to general practice, such as flexibility with appointment times or prescription collection.	**Ability to seek** (*7 publications)* - Knowledge of available general practice services among people with intellectual disability, their family and support workers. - Fear or anxiety around attending general practice or being examined. - People’s confidence in interacting with general practice staff, managing their own health information and making supported independent health decisions.
**Availability & accommodation** *(24 publications)* - The amount of consultation time needed to overcome communication barriers and manage the often-complex health status of people with intellectual disability. - The numbers of GPs and other health professionals in rural locations. - Length of waiting times to receive appointments, waiting-room times before appointments and ensuring physical accessibility.	**Ability to reach** *(9 publications)* - Geographical isolation. - Access to public transport when unable to drive. - Challenges with booking appointments independently or organising support workers to assist with attendance. - Alleviating the burden of frequent health- or disability-related appointments.
**Affordability** *(8 publications)* - Providing adequate renumeration for GPs to have longer appointments, bulk bill or complete a preventive health assessment.	**Ability to pay** *(9 publications)* - Levels of funding for people with intellectual disability and generally lower socio-economic status. - Costs of travel to general practice and support workers attendance.
**Appropriateness** *(30 publications)* - Patchy coordination of disability and health services by GPs. - Provision of targeted preventative health assessments. - Fragmented continuity of care (not accessing the same GP on every occasion). - Diagnostic overshadowing with GPs assuming symptoms are a consequence of a person’s disability without exploring other factors such as biological determinants. - Clinical inertia, where GPs fail to initiate or intensify therapy when evidence-based treatment goals are not achieved. - of the need for evidence-based guidelines specifically for people with intellectual disability.	**Ability to engage** *(30 publications)* - Communication challenges. - Support worker facilitation and advocacy during consults. - Patient capacity to make informed, independent health decisions. - Patient and support staff unaware of complete medical history.

### ‘Approachability’ and ‘ability to perceive’

Four publications identified factors that impacted on the approachability of general practice. Three of these specifically concerned the approachability of targeted preventative health assessments for people with intellectual disability,^1^ including one on the poor promotion of preventative health assessments in the community [42], and another on the low levels of GP awareness of these assessments [43]. The inability of clinical information systems to identify people with intellectual disability limited a general practice’s ability to invite, recall and remind people with intellectual disability to participate in health care [44].

Eleven publications identified demand-side factors that impacted on the ability of people with intellectual disability to perceive they had a need for health care at a general practice level. It was suggested that the generally poor educational status of people with intellectual disability made accessing health information more difficult for them [43, 45], and low health-literacy levels reduced their knowledge of common illnesses and of the situations in which general practice is required [9, 46–49]. Similarly, generally low health-literacy levels among families and support workers of people with intellectual disability also reduced the perception of their need for general practice care, as they frequently rely on others to highlight when such care is required [43, 48, 49]. The effect of this low health literacy was compounded by a lack of targeted health promotion for people with intellectual disability [45].

### ‘Acceptability’ and ‘ability to seek’

Twenty-eight publications identified factors that impacted on general practice acceptability among people with intellectual disability. Perceived discrimination, insensitivity or negative attitudes from GPs were identified in eight publications [45, 50–56], while a further four found there was GP confusion about their role in caring for people with intellectual disability [53, 57–59]. For example, some GPs believed that the primary provision of health care to people with intellectual disability was the responsibility of paediatricians or intellectual disability specialists. It was suggested that this confusion, or denial of responsibility, could lead to the provision of poorer quality care [58, 59]. Limited GP education and experience caring for people with intellectual disability was highlighted as a barrier in 25 publications [32, 42, 43, 45, 50, 51, 53–55, 57, 58, 60–73]. Adjustments to care – for example, telehealth phone consults, more flexible appointment times [73], or accommodating the collection of prescriptions from reception staff without in-person consults [51] – were found to increase the acceptability of general practice among people with intellectual disability.

Seven publications identified factors that impacted on the ability of people with intellectual disability to seek general practice care. These included two that found people with intellectual disabilities fear or anxiety around attending general practice or being examined by a GP reduced their ability to seek care [43, 65]. However, confidence to interact independently with health professionals, manage their own health information and make autonomous and supported health decisions increased their ability to seek care at general practice [46], as did knowledge of the services available and the role of GPs [43, 44, 46, 48, 49]. Factors such as support workers’ and families’ knowledge of general practice services, and their ability to manage information and appointments, were important for people with intellectual disability who relied on assistance to access general practice [44, 54].

### ‘Availability and accommodation’ and ‘ability to reach’

Twenty-four publications identified factors on the supply-side that impacted on the availability and accommodation of general practice. Insufficient consultation time to overcome communication barriers and manage the often-complex health status of people with intellectual disability was identified in 18 publications [43, 45, 50, 51, 53–55, 57–60, 65, 67–69, 72–74]. Other factors included fewer GPs and allied health professionals in rural locations [43, 50, 51, 62, 75], long waiting times to receive appointments [43, 51, 70], long waiting-room periods before appointments [51, 55, 73] and physical inaccessibility [72, 73].

Nine publications identified factors that impacted on the ability to attend general practice, for example, geographical isolation [43, 50, 55, 71, 75], difficulty accessing public transport [50] or being unable to drive [45]. Other logistical aspects, such as difficulty booking appointments or organising support workers, were also found to impact negatively on attendance [43, 46, 54]. In addition, the burden of frequent health- or disability-related appointments experienced by people with intellectual disability sometimes reduced their desire, and ability, to attend general practice. Some parents chose for their children not to participate in targeted preventive health assessments because they felt over-burdened with other appointments [48].

### ‘Affordability’ and ‘ability to pay’

Factors impacting the affordability of general practice were identified in eight publications. The most common factor was the inadequate remuneration of GPs to provide longer appointments, bulk-bill people with intellectual disability or complete preventative health assessments [45, 50, 58, 60, 65, 66, 68, 76].

Nine publications identified factors that impacted on the ability to pay for general practice care. These included the generally lower socioeconomic status of people with intellectual disability [45, 49, 54], and insufficient funding for support to enable engagement with general practice [65, 75]. For example, some studies found there was not enough funding to cover the costs of support worker attendance [70, 72], or for travel to attend general practice [50, 51].

### ‘Appropriateness’ and ‘ability to engage’

Thirty publications identified factors that impacted on the appropriateness of general practice. Inadequate care coordination by GPs – often due either to limited knowledge of relevant allied health, disability or specialist medical services, or to patchy liaison with these services – was identified in 15 publications [43, 45, 50–52, 56–58, 64, 65, 68, 69, 72, 76, 77]. Inadequate continuity of care was also common, meaning people with intellectual disability attended appointments with different GPs on different occasions. This negated the benefits of an ongoing doctor–patient relationship, and reduced the appropriateness of general practice services. Fragmented continuity of care often occurred due to GP availability issues, or a preference by support workers or residential facilities for particular GPs [52, 58, 65, 68]. Diagnostic overshadowing (incorrectly attributing symptoms to disability) [51], clinical inertia (failure to initiate or intensify therapy when evidence-based treatment goals are not achieved) [77] and a lack of intellectual disability-specific evidence-based guidelines for best practice [44] were other important factors. Finally, 10 publications identified the provision of targeted preventative health assessments as a factor increasing general practice appropriateness, as they facilitated effective medical history-taking and record-keeping, increased patient involvement and prompted medical action and follow-up [9, 42, 52–54, 56, 61, 74, 78, 79].

Similarly, 30 publications identified factors that impacted on the ability to engage with general practice. Communication difficulty, for example, was identified in 28 publications and was often due to problems with comprehension or expression or the unmet need for communication aids. GPs expressed concerns that communication difficulty reduced effective history-taking and made it hard to inform people with intellectual disability about medical conditions or procedures. This, in turn, reduced the ability of people with intellectual disability to make informed decisions and give informed consent [9, 43, 45, 46, 49–54, 56–62, 65–68, 72, 73, 76, 80–83]. Seventeen publications further identified support worker or advocate involvement during consults as a factor impacting on engagement, as they were often vital for facilitating communication and decision-making [9, 43, 49–52, 54, 56–58, 60, 64–66, 72, 73, 83]. Similarly, eight publications identified that a person with intellectual disability capacity to make independent, informed health decisions increased their engagement, but often relied on facilitation and empowerment by support workers or advocates, or the use of tools for record-keeping and advocacy [43, 46, 52, 57, 63, 72, 73, 80]. Finally, incomplete records of a medical history, held either by the person with intellectual disability or their support worker, reduced their ability to engage with general practice. This often occurred due to people with disability being accompanied by a different support worker at each appointment [9, 43, 52, 60, 61, 73, 83].

## Discussion

Ensuring that people with intellectual disability have access to general practice will continue to be an important goal for the Australian health system, and health systems internationally. Commonly reported supply-side factors identified in our review were: the level of intellectual disability-specific education or experience among GPs, whether there was sufficient consultation time for people with intellectual disability, the adequacy or otherwise of service coordination by GPs, and the provision of targeted health assessments for people with intellectual disability. The most frequently reported demand-side factors included: the ability of GPs and people with intellectual disability to communicate effectively, the involvement of support workers during consultations, the level of health literacy among people with intellectual disability and their support networks, and the knowledge of relevant medical history of people with intellectual disability, both by the client and their support workers.

Although factors on both the demand- and supply-side were uncovered, our review identified that demand-side factors – which impact the ability of patients and populations to access services – receive less attention. This is despite contemporary evidence about the need for patient-focused care and for interventions aimed at improving access to target not only the availability of general practice, but also the level of awareness and ability to access these services in the intellectual disability community. Our finding of relatively less attention to demand-side factors impacting on access has been identified by other authors when examining access barriers in other settings and contexts (not intellectual disability specific) [34, 37].

Several factors identified by our review are congruent with international studies examining primary care access barriers for people with intellectual disability. For example, communication difficulty between GPs and people with intellectual disability [13, 14, 22], limited intellectual disability-specific education or experience among GPs [13, 14, 22], insufficient time in consults [13, 14, 17, 21, 24–27, 30, 58] and inadequate continuity of care or coordination of services [12–17, 22, 26–28, 51, 58].

Some of the factors identified as impacting on access to general practice in this review are likely to be related to trends over the last four decades in reduced institutionalisation of people with intellectual disability and the development of modern paediatric services in addressing the needs of children with disability. Deinstitutionalisation has resulted in a transition away from large government-managed residential facilities and, as such, most people with intellectual disability now live in the community and access mainstream or disability-specific health, education and employment services [84–86]. Deinstitutionalisation has also resulted in a delineation of health and disability services [87], and shifted the responsibility of providing health care for people with intellectual disability away from health care staff in specialised institutions, such as medical superintendents in large residential facilities, to predominantly GPs (or specialist paediatricians) in the community [53, 56]. As a result, the provision of health care for people with intellectual disability in the general practice setting is relatively new and somewhat specialised in Australia, which may contribute to a degree of confusion reported among some GPs around their responsibility and level of involvement in providing health care for this group [53, 57–59]. Furthermore, some GPs may be inadequately experienced in caring for people with intellectual disability [43, 57, 58, 60, 68], as might other health care providers, a situation that may be compounded by a lack of intellectual disability-specific teaching in general medical education [45, 51, 60, 68, 88–90].

The utilisation of the well-cited and widely applied [36] Levesque et al. [33] framework facilitated a comprehensive and structured approach for identifying factors impacting access to general practice. It allowed for the articulation of areas requiring more emphasis in future research and policy development, such as the determinants of access on the demand-side of the framework. However, like other authors, [34, 36, 37] we found the conceptual dimensions not completely discrete, and at times it was difficult to categorise data into only one dimension of access. In many cases, factors related to multiple dimensions, and often to dimensions other than just the corresponding supply- or demand-side dimension. We found we had to continually review the definitions for each dimension provided by Levesque et al. and consider how they applied in the context of this study.

### Strengths and limitations

A strength of our review was the rigorous process of two reviewers independently conducting screening, full-text review and data extraction, and reaching consensus at every stage. The findings of this review should be viewed in light of the majority of the included publications being authored by one clinical and research expert (NL), many of which focussed specifically on one particular targeted preventive health assessment tool [53]. Selection bias may occur in studies of preventative health assessments, as GPs included in the studies are motivated and passionate about improving the health of people with intellectual disability, which may not be generalisable for all GPs [53]. Furthermore, the findings of this review may not be applicable to all people with intellectual disability in Australia. None of the included publications specified the participation of people who identify as Aboriginal or Torres Strait Islander, as culturally or linguistically diverse, as LGBTIQ+ community or who live in remote areas. Only two publications were set solely in a regional setting. Given that members of these communities may experience additional factors impacting their access to general practice [91–93], further research is required to explore the effect of compounding disadvantage.

Telehealth has been widely acknowledged as a method to improve access to health care, and its use has rapidly expanded during the COVID-19 pandemic [94, 95]. Despite this there was a lack of literature in our review that examined the utilisation of telehealth for people with intellectual disability in general practice and barriers to access. This is an area for future research.

This review is timely as it is less than a year since the 2021 launch of the National Roadmap for Improving the Health of People with Intellectual Disability [96] an Australian government policy initiative aimed at addressing health inequity experienced by people with intellectual disability. Drawing on our findings, Table 3 proposes strategies on both demand- and supply-sides to enhance access to general practice for people with intellectual disability.

**Table 3:** Strategies to enhance access to general practice for people with intellectual disability

**Demand-side** • Create Easy Read materials aimed at increasing people with intellectual disability’s understanding of services at general practice, the importance of preventive care, regular preventive health assessments and treatment of common health conditions. • Improve communication resources and provide communication training for GPs and other staff caring for people with intellectual disability specifically for the purpose of addressing demand-side limitations on access. • Strengthen training for support workers, family and allies regarding empowering and advocating for people with intellectual disability, facilitating independent decision-making and maintaining personal medical records. • Raise awareness of the availability and importance of general practice among people with intellectual disability, their families and support networks, and increase the advertising of specific services, such as targeted preventative health assessments. • Strengthen health literacy among all people with intellectual disability, their families and support workers. **Supply-side strategies** • Offer adequate remuneration for GPs providing tailored health care, including longer appointments or out-of-hours follow-up. • Promote implementation by general practice of targeted preventive health assessments and associated follow-up care through raising awareness amongst GPs and general practice staff. • Strengthen integration of allied health, disability and specialist medical services with general practice, and improve communication between these services. • Ensure general practices are physically accessible and suitable for people with intellectual disability, for example, by offering quiet spaces, shortened waiting room periods or flexible appointment times. • Upgrade clinical information systems to enable identifying, recalling and reminding people with intellectual disability to attend general practice, including specifically for preventive health assessments and follow-up care. • Improve the nature and extent of intellectual disability content during medical education and training.	

## Conclusions

This scoping review provides an overview of the research on demand- and supply-side factors that influence access to general practice for people with intellectual disability in Australia. It finds a supply-side dominance in much of the literature and suggests that interventions to increase access must target both supply- and demand-side factors to maximise effectiveness. The findings offer a basis for a balanced portfolio of strategies to enhance access to general practice that address both aspects and can support recent policy initiatives.

## Supplementary Information

**Additional file 1.**

## Data Availability

Further details on studies included in this scoping review can be retrieved by contracting the corresponding author at jodie.bailie@sydney.edu.au.

## Abbreviations

GP: General Practitioner
JBI: Joanna Briggs Institute
PRISMA-ScR: Preferred Reporting Items for Systematic Reviews and Meta-Analyses Extension for Scoping Reviews

## Footnotes

1 Structured targeted preventive health assessments, a feature of health policies both in Australia and internationally, are an approach to addressing gaps in preventive care for people with intellectual disability. They are best seen as an implementation vehicle to support the delivery of evidence-based preventive health care. In Australia, these targeted preventive health assessments for people with intellectual disability are delivered in general practice and are rebated by the Medicare Benefit Schedule (MBS) item 701, 703, 705, 707 annual time-based health assessments
