# Supplementary material for: Access to general practice for people with intellectual disability in Australia: a systematic scoping review
Source: BMC Prim Care. 2022 Nov 29;23:306. doi: 10.1186/s12875-022-01917-2 (PMC9707181; doi:10.1186/s12875-022-01917-2)
Supplement: Supplementary file 1 — Additional file 1. [file 12875_2022_1917_MOESM1_ESM.docx]

# **Supplementary file 1: Complete MEDLINE search strategy**

Database: Ovid MEDLINE(R) ALL <1946 to January 28, 2022>

Search Strategy^[[1]](#footnote-1)^:

--------------------------------------------------------------------------------

1 exp Intellectual Disability/ (101111)

2 intellectual disab*.mp. (68826)

3 Developmental Disabilities/ (21621)

4 developmental disab*.mp. (25243)

5 Learning Disabilities/ (14471)

6 learning disab*.mp. (19047)

7 mental retardation*.mp. (30380)

8 Fragile X Syndrome/ (5358)

9 fragile x syndrome*.mp. (7043)

10 mental* handicap*.mp. (2846)

11 1 or 2 or 3 or 4 or 5 or 6 or 7 or 8 or 9 or 10 (159699)

12 exp Primary Health Care/ (179348)

13 Primary health care*.mp. (102534)

14 Health Services/ (26383)

15 Health service*.mp. (471688)

16 Health Services, Indigenous/ (3746)

17 Indigenous health service*.mp. (161)

18 Aboriginal health service*.mp. (109)

19 Community controlled health*.mp. (303)

20 Preventative medicine*.mp. (347)

21 preventive medicine/ or preventive psychiatry/ (12270)

22 preventative psychiatr*.mp. (8)

23 primary* care.mp. (132118)

24 exp General Practice/ (77087)

25 (general practi* or family practi* or GP service*).mp. (141863)

26 family practi*.mp. (71091)

27 physicians, family/ or physicians, primary care/ (20886)

28 family physician*.mp. (15630)

29 primary care physician*.mp. (21335)

30 GP.tw. (45296)

31 family doctor*.mp. (4956)

32 Community Health Workers/ (6087)

33 community health work*.mp. (9029)

34 community health services/ or community health nursing/ (51359)

35 community health service*.mp. (33827)

36 community health nurs*.mp. (20283)

37 village health work*.mp. (390)

38 lay health work*.mp. (446)

39 Health Promotion/ (78653)

40 health promot*.mp. (106996)

41 community health promot*.mp. (490)

42 allied health personnel/ or licensed practical nurses/ or nursing assistants/ (16841)

43 Primary Care Nursing/ (547)

44 primary care nurs*.mp. (1385)

45 exp Nurse Practitioners/ (18660)

46 nurs* practitioner*.mp. (24375)

47 12 or 13 or 14 or 15 or 16 or 17 or 18 or 19 or 20 or 21 or 22 or 23 or 24 or 25 or 26 or 27 or 28 or 29 or 30 or 31 or 32 or 33 or 34 or 35 or 36 or 37 or 38 or 39 or 40 or 41 or 42 or 43 or 44 or 45 or 46 (964286)

48 Barrier*.mp. (362310)

49 exp Health Services Accessibility/ (120882)

50 access*.mp. (655700)

51 utili*.mp. (997974)

52 facilitat*.mp. (613559)

53 enable*.mp. (442203)

54 usab*.mp. (27832)

55 usage*.mp. (113793)

56 use*.mp. (9543322)

57 exp Health Services Research/ (181763)

58 health service* research*.mp. (43944)

59 (health service* need* and demand*).mp. [mp=title, abstract, original title, name of substance word, subject heading word, floating sub-heading word, keyword heading word, organism supplementary concept word, protocol supplementary concept word, rare disease supplementary concept word, unique identifier, synonyms] (54972)

60 need* assessment*.mp. (37477)

61 evaluat*.mp. (4325781)

62 "delivery of health care".mp. (125005)

63 treatment* barrier*.mp. (615)

64 approachab*.mp. (1000)

65 acceptab*.mp. (204440)

66 availabilit*.mp. (282533)

67 accommodat*.mp. (61265)

68 affordab*.mp. (34455)

69 appropriat*.mp. (654146)

70 "ability to perceive".mp. (1322)

71 "ability to seek".mp. (160)

72 "ability to reach".mp. (866)

73 "ability to pay".mp. (850)

74 "ability to engage".mp. (1153)

75 48 or 49 or 50 or 51 or 52 or 53 or 54 or 55 or 56 or 57 or 58 or 59 or 60 or 61 or 62 or 63 or 64 or 65 or 66 or 67 or 68 or 69 or 70 or 71 or 72 or 73 or 74 (13646324)

76 exp Australia/ (159788)

77 (Australia or New south wales or Western Australia or South Australia or Northern territory or Queensland or Tasmania or Victoria or Australian Capital Territory).mp. (207829)

78 (NSW or WA or SA or NT or QLD or TAS or VIC or ACT).tw. (400092)

79 76 or 77 or 78 (599613)

80 11 and 47 and 75 and 79 (372)

***************************

# **Supplementary file 2: Data extraction tool**

Table S2. Data extraction template based on data extraction tool within Covidence^TM^

| General information | |
| --- | --- |
| Title: | (insert text) |
| Author(s): | (insert text) |
| Publication year: | (insert text) |
| Study type:   - Empirical - Non-empirical | (select one) |
| Study design:   - Qualitative - Quantitative - Mixed-methods research - Scoping review - Commentary - Randomised controlled trial - Non-randomised trial - Case report - Cohort study - Case-control study - Systematic review - Prevalence study | (select one) |
| Methods used:   - Focus groups - Interviews - Survey - Direct observation - Case reports - Document review - N/A | (select one) |
| Author stated aim/objective: | (insert text) |
| Study content | |
| Primary health care setting:   - Government-managed general practice - Fee-for-service general practice - Aboriginal Community Controlled Health Service - Not specified | (select all that apply) |
| Jurisdiction:   - ACT - NSW - NT - QLD - SA - TAS - VIC - WA - National - Not specified | (select all that apply) |
| Rurality:   - Urban - Rural - Remote - Not specified | (select all that apply) |
| Intersectionality:   - Aboriginal and Torres Strait Islander - Culturally and linguistically diverse - LGBTQ+ - Not specified | (select all that apply) |
| Factors impacting access as defined by Levesque *et al.* | |
| Dimensions identified:   - Approachability - Ability to perceive - Acceptability - Ability to seek - Availability and accommodation - Ability to reach - Affordability - Ability to pay - Appropriateness - Ability to engage | (select all that apply) |
| Approachability (Relates to various factors such as transparency, information regarding available treatments and services and outreach activities could contribute to make the services more or less approachable) | (insert text) |
| Ability to perceive (Determined by such factors such as health literacy, knowledge about health and beliefs related to health and sickness) | (insert text) |
| Acceptability (Relates to cultural and social factors. For example, is the service culturally appropriate) | (insert text) |
| Ability to seek (Relates to the concepts of personal autonomy and capacity to choose to seek care, knowledge about health care options) | (insert text) |
| Availability and accommodation (Relates to can the service be reached physically and in a timely manner) | (insert text) |
| Ability to reach (Ability to reach health care relates to the notion of personal mobility and availability of transportation) | (insert text) |
| Affordability (Relates to cost for services and other expenses) | (insert text) |
| Ability to pay (Describes the capacity to generate economic resources to pay for health care. Poverty, social isolation, or indebtedness would be examples of factors restricting the capacity of people to pay for needed care) | (insert text) |
| Appropriateness (This dimension relates to the fit between services and client’s needs – provision of quality of care) | (insert text) |
| Ability to engage (Relates to the participation and involvement of the client in decision-making and treatment decisions, which in turn is strongly determined by capacity and motivation to participate in care and commit to its completion) | (insert text) |

# **Supplementary file 3: Details of included publications**

Table S3. Details of included publications and Levesque et al. (33) dimensions impacted by factors identified

|  | Levesque *et al.* dimensions of access impacted by identified factors | | | | | | | | | |  |  |  |  |  |
| --- | --- | --- | --- | --- | --- | --- | --- | --- | --- | --- | --- | --- | --- | --- | --- |
| Author(s) & Year | Study design & methods | Jurisdiction | Rurality | General practice setting^ | Intersect-ionality* | Approach-ability | Ability to perceive | Accept-ability | Ability to seek | Availability and accommodation | Ability to reach | Afford-ability | Ability to pay | Appropr-iateness | Ability to engage |
| Bailie *et al.*, 2021 (44) | Commentary; N/A | Not specified | Not specified | Not specified | Not specified | x | x |  | x | x |  |  |  | x |  |
| Brolan *et al.*,  2011 (60) | Commentary; N/A | Not specified | Not specified | Not specified | Not specified |  |  | x |  | x |  | x |  | x | x |
| Burton & Walters, 2013 (43) | Qualitative; Interviews | SA | Urban; Regional | Not specified | Not specified | x | x |  | x | x | x |  |  | x | x |
| Byrne *et al.*, 2015 (80) | Quantitative; Document review (including audit) | QLD | Not specified | Not specified | Not specified |  |  |  |  |  |  |  |  |  | x |
| Carrington *et al.*, 2014 (46) | Qualitative; Interviews | QLD | Not specified | Not specified | Not specified |  | x |  | x |  | x |  |  | x | x |
| Cook & Lennox, 2000 (58) | Qualitative; Survey | National | Not specified | Not specified | Not specified |  |  | x |  | x |  | x |  | x | x |
| Vanny *et al.*, 2008 (76) | Commentary; N/A | Not specified | Not specified | Not specified | Not specified |  |  |  |  |  |  | x |  | x | x |
| Eastgate & Lennox, 2003 (57) | Commentary; N/A | Not specified | Not specified | Not specified | Not specified |  |  | x |  | x |  |  |  | x | x |
| Gordon *et al.*, 2012 (78) | Quantitative; Administrative data | QLD | Urban | Not specified | Not specified |  |  |  |  |  |  |  |  | x |  |
| Beange, 1996 (45) | Commentary; N/A | NSW | Not specified | Not specified | Not specified | x | x |  | x | x | x | x | x | x | x |
| Iacono *et al.*, 2004 (51) | Qualitative; Interviews, focus groups | NSW; SA; VIC | Regional | Not specified | Not specified |  |  | x |  | x |  |  | x | x | x |
| Iacono *et al.*, 2003 (50) | Qualitative; Survey | VIC | Urban; Regional | Not specified | Not specified |  |  | x |  | x | x | x | x | x | x |
| Koritsas *et al.*, 2012 (42) | Quantitative; Administrative data | National | Not specified | Not specified | Not specified | x |  |  |  |  |  |  |  | x |  |
| Lennox & Eastgate, 2004 (52) | Case report; Case reports | Not specified | Not specified | Not specified | Not specified |  |  | x |  |  |  |  |  | x | x |
| Lennox & Taylor, 2008 (82) | Letter to Editor; N/A | Not specified | Not specified | Not specified | Not specified |  |  | x |  |  |  |  |  |  | x |
| Lennox *et al.*, 2001 (53) | Mixed-methods; Survey, literature review | Not specified | Not specified | Not specified | Not specified |  |  | x |  | x |  |  |  | x | x |
| Lennox *et al.*, 2012 (63) | Randomised controlled trial; Survey | QLD | Not specified | Not specified | Not specified |  |  | x |  |  |  |  |  |  | x |
| Lennox *et al.*, 2004 (81) | Study development; Consultation and pilot | Not specified | Not specified | Not specified | Not specified |  |  |  |  |  |  |  |  |  | x |
| Lennox *et al.*, 2016 (74) | Randomised controlled trial; Document review (including audit) | QLD | Not specified | Not specified | Not specified |  |  |  |  | x |  |  |  | x |  |
| Lennox *et al.*, 2013 (54) | Qualitative; Interviews | QLD | Not specified | Not specified | Not specified |  |  | x | x | x | x |  | x | x | x |
| Lennox *et al.*, 2010 (79) | Qualitative; Document review (including audit) | QLD | Urban | Not specified | Not specified |  |  |  |  |  |  |  |  | x |  |
| Lennox *et al.*, 2007 (9) | Randomised controlled trial; Document review (including audit) | QLD | Not specified | Not specified | Not specified |  | x |  |  |  |  |  |  | x | x |
| Lennox *et al.*, 2000 (64) | Commentary; N/A | NSW | Not specified | Not specified | Not specified |  |  | x |  |  |  |  |  | x | x |
| Lennox *et al.*, 2000 (59) | Qualitative; Survey | National | Not specified | Not specified | Not specified |  |  | x |  | x |  |  |  |  | x |
| Lennox *et al.*, 1997 (65) | Qualitative; Survey | Not specified | Not specified | Not specified | Not specified |  | x | x | x |  |  | x | x | x | x |
| Lennox *et al.*, 2006 (61) | Quantitative; Document review (including audit) | TAS | Not specified | Not specified | Not specified |  |  | x |  |  |  |  |  | x | x |
| Millar *et al.,* 2004 (62) | Qualitative; Survey | NSW | Urban; Regional | Not specified | Not specified |  | x | x |  | x |  |  |  |  | x |
| Newton & McGillivray, 2019 (55) | Qualitative; Interviews | NSW; VIC; WA | Urban; Regional | Not specified | Not specified |  |  | x |  | x | x |  |  | x |  |
| Ouellette-Kuntz, 2007 (66) | Commentary; N/A | Not specified | Not specified | Not specified | Not specified |  |  | x |  | x |  | x |  | x | x |
| Phillips *et al.,* 2004 (67) | Qualitative; Survey | VIC | Not specified | Not specified | Not specified |  |  | x |  | x |  |  |  | x | x |
| Smith & Laurence, 2021 (68) | Qualitative; Interviews | SA | Urban; Regional | Not specified | Not specified |  |  | x |  | x |  | x |  | x | x |
| Thomas *et al.*, 2011 (47) | Qualitative; Survey | WA | Not specified | Not specified | Not specified |  | x |  |  |  |  |  |  |  |  |
| Tracy, 2011 (56) | Commentary; N/A | Not specified | Not specified | Not specified | Not specified |  |  | x |  |  |  |  |  | x | x |
| Tracy & Mcdonald, 2015 (69) | Commentary; N/A | VIC | Not specified | Not specified | Not specified |  |  | x |  | x |  |  |  | x |  |
| Trollor *et al.*, 2016 (77) | Commentary; N/A | Not specified | Not specified | Not specified | Not specified |  |  |  |  |  |  |  |  | x |  |
| van Dooren *et al.,* 2013 (83) | Qualitative; Interviews | QLD | Urban | Not specified | Not specified |  |  |  |  |  |  |  |  |  | x |
| Ware & Lennox, 2016 (48) | Quantitative; Individual data meta-analysis | QLD | Not specified | Not specified | Not specified |  | x |  |  |  | x |  |  |  |  |
| Wark *et al.*, 2017 (71) | Qualitative; Focus groups | NSW; QLD | Urban; Regional | Not specified | Not specified |  |  | x |  | x | x |  |  | x |  |
| Wark *et al.*, 2015 (75) | Qualitative; Interviews | NSW; QLD | Regional | Not specified | Not specified |  |  |  |  | x | x |  | x |  |  |
| Wark *et al.,* 2014 (70) | Qualitative; Survey | NSW | Not specified | Not specified | Not specified |  |  | x |  | x |  |  | x |  |  |
| Weise *et al.*, 2017 (32) | Quantitative; Document review (including audit) | National | Not specified | Not specified | Not specified |  |  | x |  |  |  |  |  |  |  |
| Weise *et al.*, 2017 (72) | Qualitative; Document review (including audit) | Not specified | Not specified | Not specified | Not specified |  |  | x |  | x |  |  | x | x | x |
| Weise *et al.*, 2016 (49) | Qualitative; Document review (including audit) | Not specified | Not specified | Not specified | Not specified |  | x |  | x |  |  |  | x | x | x |
| Ziviani *et al.*, 2004 (73) | Qualitative; Interviews | QLD | Urban | Not specified | Not specified |  |  | x |  | x |  |  |  |  | x |
| TOTAL | | | | | | 4 | 11 | 28 | 7 | 24 | 9 | 8 | 9 | 30 | 30 |

Notes: ^ General practice setting included fee-for-service general practice, government-managed general practice and Aboriginal Community Controlled Health Services

* Intersectionality includes people with intellectual disability who also identify as Aboriginal or Torres Strait Islander, LGBTIQ+ or culturally and linguistically diverse.

NSW, New South Wales; QLD, Queensland; SA, South Australia; TAS, Tasmania; VIC, Victoria; WA, Western Australia

# **Supplementary file 4: Characteristics of included publications**

Table S4. Characteristics of included publications and Levesque et al. (33) dimensions impacted by factors identified

| **Characteristics** | **Number of publications** |
| --- | --- |
| *Rurality* |  |
| Urban | 10 |
| Regional | 8 |
| Remote | 0 |
| Not specified | 32 |
| *Jurisdiction* |  |
| Australian Capital Territory | 0 |
| New South Wales | 8 |
| Northern Territory | 0 |
| Queensland | 13 |
| South Australia | 3 |
| Tasmania | 1 |
| Victoria | 5 |
| Western Australia | 2 |
| National | 4 |
| Not specified | 14 |
| *General practice setting* |  |
| Fee-for-service general practice | 0 |
| Government managed general practice | 0 |
| Aboriginal Community Controlled Health Service | 0 |
| Not specified | 44 |
| *Study Design* |  |
| Qualitative | 21 |
| Quantitative | 6 |
| Commentary | 10 |
| Case report | 1 |
| Letter to editor | 1 |
| Mixed-methods | 1 |
| Randomised controlled trial | 3 |
| Study development | 1 |
| *Methods* |  |
| Interviews | 9 |
| Document review (including audit) | 8 |
| Survey | 10 |
| Administrative data | 2 |
| Case reports | 1 |
| Literature review | 1 |
| Consultation and pilot | 1 |
| Individual data meta-analysis | 1 |
| Focus groups | 2 |
| N/A (commentary or letter to editor) | 11 |

Note: Numbers in the rurality and jurisdiction categories do not add to 44 as some publications covered multiple rurality’s and jurisdictions.

1. Intellectual Disability was searched as a MeSH term which covers Down Syndrome, De Lange Syndrome, Cri-du-Chat Syndrome, Mental Retardation, X-linked, Prader-Willi Syndrome, Rubinstein-Taybi Syndrome, Trisomy 13 Syndrome, WAGR Syndrome and Williams Syndrome. [↑](#footnote-ref-1)
